# A New Tool to Trace Genetic Susceptibility

**DOI:** 10.1289/ehp.115-a26

**Published:** 2007-01

**Authors:** Angela Spivey

Just like individual people, various mouse strains come in different sizes and colors, and with different predispositions to disease. Since more than 99% of human genes can be matched to analogous mouse genes that have similar functions, scientists often study mouse models to better understand human disease. It’s already well known which strains of mice are genetically at greater risk for certain diseases. But what isn’t so clear is which small genetic variations, or single-nucleotide polymorphisms (SNPs), lead to these differences.

SNPs aren’t large mutations but normal variations of one letter of the genetic code. For example, a sequence that reads AATTC-CG in one strain of mouse may show up as AATACCG in another. Now Scientists who study mice as models for human disease have a new tool to help them more quickly home in on the SNPs that matter when it comes to disease susceptibility. With the completion of the NIEHS-funded Resequencing and SNP Discovery Project, announced in October 2006, researchers now have free access to a data set of 8.3. million SNPs housed on the National Center for Biotechnology Information website at http://www.ncbi.nlm.nih.gov/SNP/.

Researchers at Perlegen Sciences, under contract to the National Toxicology Program, resequenced the genomic DNA of 15 genetically diverse research mouse strains by comparing the sequences to a reference mouse strain (the first mouse strain to have its DNA completely sequenced to very high accuracy). Kelly Frazer, principal investigator of the project and vice president of genomics at Perlegen Sciences, says, “We take these pieces of DNA and align them to that reference sequence, and we look for differences.” To do that, the scientists used the same high-density oligonucleotide array technology that was used to discover common DNA variations in the human genome.

The completion of the project provides a powerful tool; many discoveries should come when scientists use it. “We’re looking forward to researchers using these . . . SNPs in comparative studies of the different mice strains that are physiologically and physically well characterized,” Frazer says.

David Threadgill, an associate professor of genetics at the University of North Carolina at Chapel Hill, says the data set was critical in recent studies that revealed genes that may contribute to human susceptibility to liver damage from acetaminophen. His work, currently under review for publication, was inspired by a paper published 5 July 2006 in *JAMA* showing that a subset of the study participants showed greater susceptibility to liver damage from taking recommended doses of acetaminophen. “However, clearly it’s unethical to go in and treat thousands of individuals to try to find the underlying genetic causes of that,” Threadgill says.

Instead, Threadgill and colleagues used the SNP maps of the 15 mouse strains to identify genetic variance between strains of mice that differed in liver toxicity due to acetaminophen. They then tested two candidate genes in a small human study. They found that variance in the two candidate genes correctly predicted the patients who showed high levels of serum markers of liver toxicity after taking the maximum recommended dose of acetaminophen for several days. **“**Without these detailed SNP maps, we would not have been able to have very quickly done that translational test, which we were able to do just in a matter of months,” Threadgill says.

NIEHS director David Schwartz says the conclusion of the Resequencing and SNP Discovery Project represents a milestone that builds on fundamental accomplishments such as the 2003 completion of the human genome sequence. “This accomplishment provides the tools to discover genes in mice that play critical roles in the response to environmental toxicants and the development of disease,” Schwartz says. “The environmentally responsive genes and the disease-related genes in mice are very likely to be important in human disease. This development can be used to identify those at risk, prevent the development of disease, and also identify promising new forms of treatment.”

